# Honor Bright

**Published:** 1879-01

**Authors:** Ben. H. Pratt


### THE BISTOURY.

Vol. XIV. ELMIRA, N. Y., JANUARY, 1879. No. 4.

### Contributions.

### HONOR BRIGHT.

BEN. H. PRATT.

Unobtrusiveness, sobriety, honesty, virtue, faithfulness, industry, continuance in well doing—these, in men, the world never ceases to honor, theoretically.

In public addresses, in sermons, in more formal conversations, in labored articles of a moralistic turn, it always sounds well to bestow much splendor of rhetoric upon modest men, and to condemn impudence unqualifiedly. Public assemblies never fail to applaud eulogistic reference to "modest merit;" affect to see all charms consistent with manly worth in him who vaunteth not himself, is not puffed up, and endures in repineless silence his unrecognized abilities. "Modest Merit!" So trite is this saying, that he of a retiring disposition, who never thrusts himself forward in the push for place and preference, is ever recognized as much worthier than he who clamors for them.

It is popular to boast, of one's friend, that he is strictly temperate. The hearer never disputes the assertion that there is no higher claim upon society than he possesses who never exceeds the boundaries of sobriety.

"Honesty is the best policy." "An honest man's the noblest work of God." These maxims are in the mouth of all. They are accepted as axiomatic—no more needful of proof than that the whole is greater than one of its parts.

"Virtue is its own reward." "Be virtuous and you will be happy." Everybody admits the truth of these sentiments. The vilest man on earth won't ask you to prove them. The harlot utters them tripplingly upon her tongue.

The faithful man, he that guards his neighbor's or his employer's, or his client's or his friend's trust, as though it were his own, is commended by all who take occasion to speak of him. Faithfulness is the best of recommendations.

The industrious man or woman, who has no time to bestow upon other's business, is admiringly viewed by all. "The hand of the diligent maketh rich." People can't praise such an one enough.

Adherence to a strictly honorable policy, through good and evil report, entitles one to such plaudits from his fellow men as would seem to doubly repay all the sacrifice made in the pursuit of that policy.

The above sentiments are those of every-day repetition. They are the stock in trade of the moralizer, the preacher, the writer, the conversationalist. They are life axioms, or statements which require no proof, because Society admits their truth without argument. Nobody disputes them, because, in doing so, he would run counter to the opinions of too large a number—because he must contend against custom so old that the memory of man runneth not to the contrary—because a denial of them would sap the whole foundation of our social and moral economy—because what has been held as truth so long, must be truth, and is accepted as such without question—because it sounds well, and——well for fifty reasons which might, but need not, be enumerated.

We wish to remark, textwise, that, theoretically, the world honors such sentiments; practically, it doesn't do any such thing. Let us see what it does do.

A young man, respectably born into, or otherwise properly projected upon a cummunity, having absorbed the sentiments embraced in the foregoing paragraphs, and concluding to avail himself of their glowing promise, enters upon his studies, his trade, his profession, his situation or occupation, of whatever sort it may be, and clings firmly to his purpose of being correct in every particular.

Temperate, virtuous, honest, faithful, diligent, persistent, unassuming, he works away, hopeful of the promised rewards, yet patient under their slow coming. He is associated, necessarily, both in business and society, with young men of good, bad and indifferent principles and habits. He has ample opportunities for noting the success or failure of those about him, and also for getting at the causes thereof, direct and indirect. He is thoughtful, observing, discriminative, and means to be just in his conclusions from all the premises at his command. He does not purpose to test the truth of the sentiments referred to, but to trace their workings in the cases of those who come within his own experience and observation. He does not endeavor to prove their fallacy, for he is anxious that all may be true of them which has been preached. He simply applies fact and theory, one to the other, hoping to find them coincident. Let's see what he finds.

A young man in a bank; friend of our observer; more or less an associate; good fellow; moderate abilities; socially inclined; acquires, by degrees, unsteady habits; spends late hours convivially; becomes addicted to drink; is remonstrated with by his employer; temporarily reforms, then falls into old habits; becomes a debauche and loses his position. "What a pity!" cries all his acquaintances. "Too bad, poor fellow, with only one bad habit, that he should be thrust out of a place and into ruin! Now he'll go to the dogs, sure. He's so completely discouraged, that he can't make an effort to reform." Everybody at once takes a great interest in him. People who knew him only by sight, feel it their special duty and privilege to go to him and endeaver to win him back to sobriety; and to further encourage such effort on his part, seek out and plead with his employer to restore him to his place under promise of reformation. He is restored, tried again, falls again, is again without occupation. Again the community makes outcry of vast pity. Again society bands itself together in a league to rescue this young man, and again is successful in getting him into his old place, or makes a new and better one for his express benefit. He accepts the same as his due. He has begun to believe that he is too important to the community in which he lives to be allowed to drop out of its care, and thereupon assumes an indifference to their efforts or to his own prospects which is simply the outgrowth of the treatment accorded him. Again he lapses, and thenceforward he has become the pitied and petted of his townsfolk, without exception. Though longer unfitted to hold place of trust among them, he is still the object of special care and special commiseration, pointed out as that poor fellow who is his own worst enemy—brilliant young fellow with an unfortunate infatuation for drink—smart as a whip, but alas, he drinks—won't somebody invent some new plan, some grand scheme, to save him from himself!—he never tried to save himself, but, forsooth, we must expend all our powers of sympathy, and time and money to save him—he don't care for himself or for us—he wouldn't try to resist one bad habit that would redeem his character—he has but to stop making of himself a creature such as a sentient hog would be indignant at being called—but he won't do it, and so becomes the lifelong pet of the public, one whom none condemns, all pity, all strive to help, and who, should he abstain from drink a whole month, could make a selection of the best bank clerkship in his town and oust the most temperate and steadiest young man in the community. Yet men laud temperance as one of the noblest virtues and the surest road to business success, while they would at any time overlook the lifelong sobriety of a fellow citizen, bestowing upon him not one word of commendation, or afford him any encouragement to continue in the pursuit of abstinence.

Within our experience, the cases in which the self sustaining, the self-controlled, the self-helped, the self-poised young man has been compelled to give place to the self-regardless, self-destroying, are not few. The one becomes the protege of the public; the other must shift for himself. Thus the dear public becomes the apologist of debauchery, and gives no heed to the deserving. Thus society sustains in deed what it condemns in word, and that fine sentiment which it pleases itself to utter so gibly, and to which it responds so soulfully, is but a sentiment after all, a smoothly sounding phrazing of a popular fallacy. It says, "be temperate and be respected;" but it means "be drunken and be petted." It holds out good words for the encouragement of sobriety, but extends that more substantial premium for inebriety, help of the most earnest sort—help unasked—help unthanked—help unrewarded—help which is like pearls cast before swine. Society will go further. It will snatch the crust from the mouth of the deserving and hurl loaves at the spurning feet of the utterly undeserving. This is how it works.

Far be it from us to condemn the use of all the means in our power to reclaim those who have gone astray from the paths of rectitude; but to do it *ad infinitum*, *ad nauseam*, even beyond the point of hopeful reclamation, and at the expense of the worthier man, is an outrage upon decency, and holds out golden lures to an indulgence in proprofligacy. Out upon such policy! Shame upon you, business men, professed philanthropists, who eye askance your deserving associates and helps, while you raise heaven and earth to make place for those who only laugh at your efforts, or sullenly accept your proffers as though doing, rather than receiving, a favor. Shame upon you, Society, that instead of closing your doors against him who has shown himself unworthy of your countenance, and thus teaching him that he who cares not for his own respectability deserves nothing of others, you open wide your portals, aye, even your arms, to receive and reward him. There are repentant prodigals for whom the fatted calf cannot be slain and partaken of with too much rejoicing; but for the unrepentant, in whose being there dwells no gratitude for your oft-repeated efforts to lure him back into the social circle, there should be nothing save regrets. The sooner he goes his way, the sooner he will find the end thereof, and if worth reclaiming, will reclaim himself. So long as our worthiest young men are made naught of, and our scape-graces are fondled and feted and helped to all the plums in the social and commercial pie, so long will there be held out premiums for debauchery. It's bad enough for a man to make a fool of himself over liquor; let us not increase the list of fools by making of ourselves bigger fools by bowing down and worshiping his folly. Ephraim became joined to his idols, and the Authority of the universe said, "let him alone." Shall man be more lenient than his Creator? Shall we, the creatures, be more solicitous for the reclamation of our kind, than the Creator announces Himself, through inspiration, concerning him "made in His own image?"

Virtue finds itself lauded the world over. It is the expressed province of Society to give the libertine a wide berth, and Society utters noble sentiments when it discourses upon this theme. How is it with the *roue?* In what way suffers this gallant gentleman when he knocks for admission at the doors of society's brightest luminaries. Is the servant instructed to slam the door in his lascivious face? He is invited to take the easiest chair in the drawing-room. Does madam, or do her daughters, cognizant of wrongs done certain of their sex, meet him with any show of disapproval of his course? Do they exhibit it in their reception of him? Do they frown upon the villain, and give him most distinctly to understand that the man who regards the virtue of woman only a mere something to be overcome, and the victory to be boasted of, cannot associate with the darlings of society? Do anxious mammas deny their daughters the company of such men? Are our girls instructed to accept no compliments, no invitations, no gifts from the acknowledged destroyer of others' chastity? Not at all. The most licentious rake of his class, wear he the garb and manners of a gentlemen, and particularly if he have the means to support an establishment, carries with him the magic open sesame that swings wide for him the best doors in the land. Instead of repulsing such an one, and consigning him to the class with which he may harmlessly ply his vile vocation, he is the courted beau of what we are compelled to style our best society. He actually bears about with him a power which doubly fascinates those among whom he moves, and they who cannot longer appear blind to his reputation, characterize him as a sad dog, or a dreadful fellow, but dote upon him all the more. "Virtue is its own reward." Is it? "Then," says the man of policy, "no virtue for me, if you please." "Be virtuous and you will be happy." Ah? "Not any, thank you," exclaims the male butterfly, just out of the scholastic chrysalis, "I much prefer the unhappiness which I see so brilliantly explified among those with whom I am brought into daily contact." What can society expect of men when it thus openly becomes the apologist of the broadest license for men? What can the pulpit do, or the Christian Associations do, or the sunday schools do, or parental admonitions do, or the finely spun theories of moralists do, against the mighty counter-influence which society is continually exerting by proclaiming that, theoretically, virtue is the noblest trait a man can possess, but practically, the lack of it is the most fascinating lure that society men can invest themselves with?

Is honesty the best policy? Speaking, mind you as a politic measure. Do our honest men soonest become rich? Do they most certainly achieve honor, fame, distinction, by pursuing a strictly honest course of living? Do not, on the contrary, the less scrupulous in matters of business, soonest acquire that which men are pretty generally agreed upon as the good things of this life? We hear of men too honest for their own good. They are pointed out as anomalies. They are always admirable in the common speech of people, but the worldly wise, in their hearts, call them fools. Doesn't community hold out premiums for dishonesty? Does it reward honesty save in sentiment, and when it would extort the applause of men? Verily, the honest man has little business with those of this generation. Business and honesty are like religion and business—they don't work happily together under the present code. The fellow who owed everybody, and who, in that condition, "got religion" and came out strong in meeting, when it was intimated to him that now he would certainly pay his debts, hit the nail on the head exactly when he replied, "Ah, my brother, religion is religion, but business is business."

One would think that at least industry would tell in the long run. But the industrious man soon becomes known as a plodder, one who is unsocial, devotes no time to his drony friends, and though he may succed in maintaining a subsistence, it is out of all proportion to his work, as compared with the getting of those less inclined to constant exertion. It has become more popular to live by one's wits than by one's work. The world don't value the worker much these days. Industry is far below par, and meets with little encouragement.

One of the ideas that men most love to harp upon in these days, is the merit of modesty. Unfortunately, theory and practice are wide apart in this matter. The unostentatious man, who cannot push himself, puff himself, insinuate himself, roar himself, write himself, or otherwise thrust himself upon the attention of the public, can be safely said to be chock full of meritorious something, for the negative can never be proven. You can't "bring out" a modest man. You can assert the wildest impossibilities concerning him without fear of contradiction. But he never gets on with all his merit, and it somehow never avails him anything. It's a thing to applaud in others, but nobody cares for any of it for himself. "Cheek," short for impudent ignorance and importunate, irrepressible selfishness, is much preferred, and far more eagerly coveted. It achieves something soonest of any other known human characteristic. Everybody condemns cheek, and praises its opposite in man; yet all that a man hath will he give for cheek, for cheek encompasseth all, comprehendeth all, scoopeth in all. It used to be thought that to achieve success required that one merely merit it. Now about the only merit we hear of is embodied within the corporality of the modest men; and about the only hindrance to success, now, is the modesty which somehow will not release its embrace of merit. Hence it is become quite superfluous to merit anything, inasmuch as merit often stands in the way of and is an effectual barrier to the realization of that which is sought after.

In fine, the world is teaching in almost every department of life, and in every branch of living, two distinct codes, the one the antipodes of the other. Its precepts, as given through its teachers, hold up the nobility of temperance, the splendor of virtue, the beauty of sobriety, the grandeur of honesty, the sublimity of fidelity, the delights of industry, the merit of modesty; and we admire all these, purposing in our hearts to strive for their achievement. This in our premature days. Arrived at manhood, we find the world teaching by example quite the contrary, to wit: that temperance fails to inspire anything more than a feeble respect, and which avails nothing, other things being equal; that lust and lechery are rather attractive than otherwise in men about town; that men don't ask *how* one got his money, but how much there is of it; that fidelity exists only as a pretty sentiment to hang a romance upon; that industry, and all that sweat of the brow business, is rather degrading than otherwise; that modesty might as well retire into a hermitage, as there's no earthly use for it among men.

Honest, now, aren't we a two-faced, double-dealing, hypocritical world? Candidly speaking, don't our sentiments and our practice clash wickedly. Honor bright, don't we spoil all our teaching by our conduct? Don't we undo as fast as we do? Of what avail is it that we listen to ennobling sentiments from the lips of those who utter them merely to get the applause of their listeners—utter them in the consciousness that they are glittering abstractions, which ought to be the governing principles of our lives but are not? Far better were it to hold out to the young no such rewards for meritorious living, than to deceive them by promising without fulfilling. But our teachers go further than this—they actually bestow the rewards upon those who deserve them not, and withhold them from those who hardest strive to deserve them.

